# Cellulose Nanocrystals/Chitosan-Based Nanosystems: Synthesis, Characterization, and Cellular Uptake on Breast Cancer Cells

**DOI:** 10.3390/nano11082057

**Published:** 2021-08-12

**Authors:** Ricardo J. B. Pinto, Nicole S. Lameirinhas, Gabriela Guedes, Gustavo H. Rodrigues da Silva, Párástu Oskoei, Stefan Spirk, Helena Oliveira, Iola F. Duarte, Carla Vilela, Carmen S. R. Freire

**Affiliations:** 1 Department of Chemistry, CICECO—Aveiro Institute of Materials, University of Aveiro, 3810-193 Aveiro, Portugal; r.pinto@ua.pt (R.J.B.P.); nicoleslameirinhas@ua.pt (N.S.L.); gabriela.guedes@ua.pt (G.G.); gustavohrs@ua.pt (G.H.R.d.S.); ioladuarte@ua.pt (I.F.D.); 2 Department of Biology, CESAM—Centre for Environmental and Marine Studies, University of Aveiro, 3810-193 Aveiro, Portugal; parastu.oskoei@ua.pt (P.O.); holiveira@ua.pt (H.O.); 3 Institute of Bioproducts and Paper Technology, Graz University of Technology, Inffeldgasse 23, 8010 Graz, Austria; stefan.spirk@tugraz.at

**Keywords:** cellulose nanocrystals, chitosan, folic acid, fluorescein isothiocyanate, nanosystems, physical adsorption, cellular uptake, cellular exometabolomics, folate receptor-positive cancer cells

## Abstract

Cellulose nanocrystals (CNCs) are elongated biobased nanostructures with unique characteristics that can be explored as nanosystems in cancer treatment. Herein, the synthesis, characterization, and cellular uptake on folate receptor (FR)-positive breast cancer cells of nanosystems based on CNCs and a chitosan (CS) derivative are investigated. The physical adsorption of the CS derivative, containing a targeting ligand (folic acid, FA) and an imaging agent (fluorescein isothiocyanate, FITC), on the surface of the CNCs was studied as an eco-friendly methodology to functionalize CNCs. The fluorescent CNCs/FA-CS-FITC nanosystems with a rod-like morphology showed good stability in simulated physiological and non-physiological conditions and non-cytotoxicity towards MDA-MB-231 breast cancer cells. These functionalized CNCs presented a concentration-dependent cellular internalization with a 5-fold increase in the fluorescence intensity for the nanosystem with the higher FA content. Furthermore, the exometabolic profile of the MDA-MB-231 cells exposed to the CNCs/FA-CS-FITC nanosystems disclosed a moderate impact on the cells’ metabolic activity, limited to decreased choline uptake and increased acetate release, which implies an anti-proliferative effect. The overall results demonstrate that the CNCs/FA-CS-FITC nanosystems, prepared by an eco-friendly approach, have a high affinity towards FR-positive cancer cells and thus might be applied as nanocarriers with imaging properties for active targeted therapy.

## 1. Introduction

Polysaccharides are natural polymers composed of monosaccharides linked by glycosidic bonds that hold great promise as eco-friendly building-blocks to develop advanced functional materials [1]. Within the vast portfolio of polysaccharides, cellulose (i.e., linear homopolysaccharide composed of β-D-glucopyranose units linked by β-(1,4) glycosidic bonds) is amongst the most studied natural polymer in multiple fields of research [2,3,4,5]. This polysaccharide, and particularly its nanoscale forms, viz. cellulose nanocrystals (CNCs), cellulose nanofibrils (CNFs) and bacterial nanocellulose (BNC) [6], are showing incredible potential as precursors of nanomaterials for a deluge of applications, including in the fight against cancer [7,8,9]. In fact, the number of developed systems for cancer diagnosis and treatment [7,9,10] is increasing at a fast pace since this large group of diseases is the second leading cause of death globally, with ca. 10 million deaths in 2020 [11,12].

CNCs have a rod-like morphology [13] and are of particular relevance in this context. Recent studies have demonstrated that the shape of the nanocarriers is an important parameter for their efficiency, and that elongated or filamentous nanostructures present several benefits over spherical ones in terms of surface area-to-volume ratio, rate of clearance from the body and elimination mechanism, as well as an enhanced uptake rate by tumor cells [14,15,16,17]. Moreover, CNCs are rich in hydroxyl groups that enable easy coupling (covalent and non-covalent) of targeting, imaging, and therapeutic agents [9,18] to actively target cancerous cells [19].

As an example, Roman and co-workers have looked extensively into the use of CNCs as nanocarriers by the functionalization of these elongated nanostructures solely with fluorescein isothiocyanate (FITC) as an imaging agent [20,21,22] or with both FITC and folic acid (FA) as imaging and targeting agents [23,24,25], respectively. These authors showed the potential of these nanosystems for the active targeted delivery of chemotherapeutic agents to folate receptor (FR)-positive cancer cells, such as human (DBTRG-05MG, H4) and rat (C6) brain tumor cells [23], KB and human breast cancer cells (MDA-MB-468) [24,25]. Following the same idea, Raja et al. [26] chemically modified CNCs via covalent tethering of PEGylated biotin (targeting ligand) and perylenediimide (imaging agent) and demonstrated their aptness for cell labelling and imaging of fundamental cells of the immune system (J774A.1 macrophages and primary DCs), the connective tissue (NIH-3T3 fibroblasts), and a severe pathological state (HeLa adenocarcinoma cells).

Despite the promising results of the previously enumerated studies, the functionalization of the CNCs nanosystems was mostly performed via multiple synthesis steps and with fairly toxic reagents [20,21,22,23,24,25,26], and thus the need for alternative methodologies [18,27] is of utmost importance. Therefore, the use of CNCs in conjunction with a chitosan (CS, i.e., linear heteropolysaccharide obtained from chitin via *N*-deacetylation [28]) derivative with targeting and imaging ligands seems an interesting approach (inspired by their opposite surface charge) that has never been addressed before. In this scenario, the present study describes the synthesis, characterization, and cellular uptake on breast cancer cells (MDA-MB-231 cell line) of a dual polysaccharide nanosystem based on CNCs and a CS derivative with opposite surface charges. The physical adsorption of the chitosan derivative, containing targeting (FA) and imaging (FITC) ligands, on the surface of the CNCs was studied as an environmentally friendly approach to engineer CNCs nanosystems with enhanced cellular internalization by MDA-MB-231 cells. The CNCs nanosystems were characterized in terms of structure, optical properties, fluorescence, stability in simulated physiological and non-physiological conditions, morphology, in vitro cytotoxicity, cellular internalization, and extracellular metabolomic profile to evaluate their suitability for the targeting and imaging of breast cancer cells.

## 2. Materials and Methods

### 2.1. Chemicals, Materials, and Cells

Acetic acid (97.0%, Sigma-Aldrich (St. Louis, MO, USA)), chitosan (CS, purified powder, MW 15,000, >85% degree of deacetylation, viscosity >90 cPs at 1% solution, Polysciences (Warrington, PA, USA)), Azpack™ cotton wool (BP grade, Fisher Scientific (Hampton, NH, USA)), dimethyl sulfoxide (DMSO, >99%, LabScan (Bangkok, Thailand)), 3-(4,5-dimethylthiazolyl-2)-2,5-diphenyltetrazolium bromide (MTT, 98%, Sigma-Aldrich (St. Louis, MO, USA)), 1-ethyl-3-(3-dimethylaminopropyl)carbodiimide (EDC, ≥98.0%, Sigma-Aldrich (St. Louis, MO, USA)), fluorescein isothiocyanate (FITC, isomer I, 90%, Sigma-Aldrich (St. Louis, MO, USA)), folic acid (FA, 97.0%, Sigma-Aldrich (St. Louis, MO, USA)), methanol (99.9%, Fisher Chemical (Hampton, NH, USA)), phosphate buffered saline (PBS, pH 7.2, Sigma-Aldrich (St. Louis, MO, USA)), sodium hydroxide (99.0%, Fisher Chemical (Hampton, NH, USA)), and phosphotungstic acid hydrate (HPW, p.a., Carl Roth (Karlsruhe, Germany)) were used as received. Other chemicals and solvents were of laboratory grade.

RPMI 1640 medium, fetal bovine serum (FBS), L-glutamine, penicillin/streptomycin, fungizone (250 U mL^−1^) and trypsin–ethylenediaminetetraacetic acid (EDTA) (0.25% trypsin and 1 mM EDTA) were purchased from Gibco^®^ (Life Technologies, Carlsbad, CA, USA). Type 1 ultrapure water (resistivity of 18.2 MΩ cm (25 °C)) was filtered by a Simplicity^®^ Water Purification System (Merck Millipore, Darmstadt, Germany). The Spectra/Por^®^ regenerated cellulose dialysis membranes (MWCO 6–8 kDa) were purchased from Daigger Scientific (Hamilton, NJ, USA). The MDA-MB-231 breast cancer cells were obtained from the American Type Culture Collection (ATCC, Manassas, VA, USA).

### 2.2. Preparation of Cellulose Nanocrystals (CNCs)

The CNCs were prepared via acid hydrolysis with phosphotungstic acid according to a modified literature protocol [29]. In a typical procedure, 322 g of HPW (111 mmol) and water (110 mL) were placed in a 500 mL three-neck round bottom flask equipped with a reflux condenser. Then, the mixture was heated to 90 °C and thoroughly washed cellulose cotton fibers (9.0 g, repeated washing with distilled water to remove impurities) were added in small portions. To achieve hydrolysis of the cotton fibers, the mixture was stirred for 48 h under reflux. After cooling to room temperature under vigorous stirring, the mixture was transferred to a 3 L extraction funnel. Diethyl ether (500 mL) and water (250 mL) were added, resulting in the formation of three phases (heaviest phase: HPW/ethanol/water, medium phase: aqueous, upper phase: ethanol). The medium aqueous phase was separated, and the CNCs were purified by repeated centrifugation and washing with NaOH (0.1 M) and distilled water to remove traces of tungstate. The average yield after freeze-drying (5 experiments) was 86.3 ± 3.1%.

The obtained CNCs have an elemental composition of 40.3 ± 0.4% of carbon (C), 5.4 ± 0.1% of hydrogen (H) and 54.3 ± 0.5% of oxygen (O) determined by elemental analysis (LECO TruSpec 630-200-200 CHNS, LECO Corporation, Michigan, USA), dimensions of 45 ± 12 nm (diameter) and 364 ± 74 nm (length) (micrographs acquired on a HR-SEM-SE SU-70 microscope, Hitachi High-Technologies Corporation, Tokyo, Japan), and zeta (ζ)-potential of −11.5 ± 0.7 mV at pH 7 (Zetasizer Nano ZS, Malvern Panalytical, Cambridge, UK).

### 2.3. Synthesis of the FA-CS-FITC Derivative

Firstly, CS (ζ-potential of +68.4 ± 2.1 mV at pH 3) was functionalized with folic acid (i.e., CS-FA derivative) following the methodology described by Lee et al. [30] with some modifications. Briefly, EDC (20 mg) was added to an FA solution (20 mg in 10 mL DMSO) and allowed to react for 90 min at room temperature to activate the carboxylic groups of FA. This solution was then added dropwise to a CS solution (100 mg in 20 mL of 1 M acetic acid aqueous solution) under magnetic stirring, in an ice bath, and the pH was adjusted to 6 using an aqueous solution of NaOH (1 M). The reaction mixture was kept under stirring over 48 h at room temperature in the dark. The CS-FA derivative was dialyzed in distilled water for three days to remove the unreacted FA, followed by freeze-drying, which generated a yellowish solid with an elemental composition of 47.9 ± 0.4% of C, 6.2 ± 0.3% of H, 33.0 ± 0.5% of O, and 12.9 ± 0.3% of nitrogen (N), and a ζ-potential value of +57.0 ± 4.4 mV at pH 3.

The FA-CS-FITC derivative was synthesized via an adapted method described by Huang et al. [31]. A solution of FITC (12 mg in 18 mL of methanol) was added dropwise to a solution of CS-FA (60 mg in 18 mL of 1 M acetic acid aqueous solution) under magnetic stirring. The reaction mixture was allowed to react for 24 h at room temperature in the dark. Then, the pH of the solution was adjusted to 10 with an aqueous solution of NaOH (1 M). The solution containing the FA-CS-FITC derivative was dialyzed in distilled water for four days, followed by freeze-drying, which produced a light orange solid with an elemental composition of 50.0 ± 0.5% of C, 5.3 ± 0.3% of H, 37.5 ± 0.4% of O, 6.6 ± 0.1% of N and 0.6 ± 0.1% of sulphur (S), and a ζ-potential value of +51.9 ± 3.6 mV at pH 3.

### 2.4. Preparation of the CNC Nanosystems Functionalized with the FA-CS-FITC Derivative

The CNCs were functionalized with the FA-CS-FITC derivative via electrostatic assembly. Briefly, a solution of FA-CS-FITC derivative (1, 2 or 4 mg in 40 mL of 1 M acetic acid aqueous solution) was added dropwise to a suspension of CNCs (50 mg in 10 mL of ultrapure water), as summarized in Table 1. The mixture stood for 1 h under magnetic stirring in the dark. Afterwards, the suspension was centrifuged (13,400 rpm, 10 min, Megafuge 16R centrifuge (Thermo Scientific, Waltham, MA, USA)) and washed one time with 1 M acetic acid aqueous solution and three times with ultrapure water, followed by freeze-drying, which yielded solid nanosystems with an orange coloration.

### 2.5. Characterization Methods

The Fourier transform infrared-attenuated total reflection (FTIR-ATR) spectra of all samples were collected in the solid-state in a Perkin-Elmer FT-IR System Spectrum BX spectrophotometer (Perkin-Elmer, Waltham, MA, USA), coupled with a single horizontal Golden Gate ATR cell (Specac^®^, London, UK), using 32 scans at a resolution of 4 cm^−1^ in the wavenumber range of 600–4000 cm^−1^.

The optical spectra were recorded on a Thermo Scientific Evolution 220 UV-visible spectrophotometer (Thermo Fisher Scientific, Waltham, MA, USA) using 100 scans min^−1^ with a bandwidth of 2 nm and an integration time of 0.3 s in the wavelength range of 250–600 nm. The CS and the FA-CS-FITC samples were analyzed in acidic aqueous solutions (1 M of acetic acid), and the FA and FITC samples were examined in DMSO and ethanol (1 mg mL^−1^), respectively. For the solid samples, namely the CNCs and CNCs/FA-CS-FITC_3 nanosystem, a Jasco V-560 UV-visible spectrophotometer (JASCO Corporation, Tokyo, Japan) was utilized also in the wavelength range of 250–600 nm.

The fluorescence emission spectra were obtained on a Horiba Jobin-Yvon FluoroMax-4 spectrofluorometer (Horiba Jobin-Yvon, Kyoto, Japan) with a 2.0 nm width of both excitation and emission slits, and an integration time of 0.1 s in the wavelength range of 500–650 nm. The appropriate excitation wavelengths (λ_ex_) were obtained as the wavelength of maximum absorption as follows: λ_ex_ = 450 nm for the FITC sample in ethanol (1 mg mL^−1^) and λ_ex_ = 440 nm for the FA-CS-FITC in an acidic aqueous solution (ca. 1 mg mL^−1^, 1 M of acetic acid). The fluorescence spectrum of the solid CNCs/FA-CS-FITC_3 nanosystem was recorded using a Jasco spectrofluorometer FP-8300 (JASCO Corporation, Tokyo, Japan) equipped with a xenon lamp, using a scan speed of 200 nm min^−1^, bandwidth of excitation and emission of 5 nm and excitation wavelength of 495 nm, also in the wavelength range of 500–650 nm.

Zeta potential measurements were performed on a Malvern ZetaSizer Nano-ZS (Malvern Panalytical, Cambridge, UK) at room temperature and different media depending on the analyzed sample, as described above. All measurements were performed in triplicate.

Scanning transmission electron microscopy (STEM) images were acquired in a field-emission gun (FEG) SEM Hitachi SU-70 microscope (Hitachi High-Technologies Corporation, Tokyo, Japan) operated at 15 kV. Samples were prepared by placing a drop of the suspensions of CNCs and CNCs/FA-CS-FITC_3 nanosystem directly onto carbon-coated copper grids and allowing the solvent to evaporate. The size (diameter and length) of the samples was determined by measuring over 100 rods (elongated nanostructures) for each sample, using the Fiji image processing software.

### 2.6. Stability Tests

The stability of the CNCs/FA-CS-FITC nanosystems was evaluated in simulated non-physiological and physiological conditions, namely at pH 2.1 (aqueous solution of 0.01 M HCl) and at pH 7.2 (PBS), for 24 h and 48 h [32]. Typically, 1 mg of CNCs/FA-CS-FITC was added to vials containing 2 mL of the two media. Then, the suspensions were placed on an orbital shaker at 37 °C in the dark for 24 h and 48 h. After these periods, the suspensions were centrifugated at 13,400 rpm during 5 min (Megafuge 16R centrifuge, Thermo Scientific, Waltham, MA, USA). The absorbance spectra of the corresponding supernatants were recorded on a Thermo Scientific Evolution 220 UV-visible spectrophotometer (Thermo Scientific, Waltham, MA, USA) to quantify the amount of the derivative that was released (calibration curve: $y=2.2591x-0.0141,\;R^{2}=0.9995$), concentration range of the FA-CS-FITC derivative: 0.005–0.5 mg mL^−1^).

### 2.7. Cell Culture

MDA-MB-231 cells were cultured in RPMI culture medium with L-glutamine, without folic acid, supplemented with 10% FBS, 2 mM L-glutamine, 1% penicillin-streptomycin (10,000 U mL^−1^), and 1% fungizone (250 U mL^−1^), at 37 °C in a humidified atmosphere with 5% CO_2_. Cells were daily observed for confluence and morphology using an inverted phase-contrast Eclipse TS100 microscope (Nikon, Tokyo, Japan). Sub-confluent cells were trypsinized with trypsin-EDTA (0.25% trypsin, 1 mM EDTA) when monolayers reached 70% of confluence.

### 2.8. In Vitro Cytotoxicity Assay

Cells were seeded in a 96-well plate at 20,000 cells/well and, after cell adhesion, the cell culture medium (in the 96 well plates) was replaced with fresh medium containing CNCs and CNCs/FA-CS-FITC nanosystems at 0, 12.5, 25, 50, 100, 200 µg mL^−1^ and then further incubated for 24 h at 37 °C, 5% CO_2_ humidified atmosphere.

At the end of the exposure time, 50 µL of MTT solution (1 mg mL^−1^ in PBS pH 7.2) were added to the medium and cells were incubated for 4 h. After that, the culture medium with MTT was removed and replaced by 150 μL of DMSO and the plate was placed in a shaker for 2 h in the dark to completely dissolve the formazan crystals. The absorbance of the samples was measured with a BioTek Synergy HT plate reader (Synergy HT Multi-Mode, BioTeK, Winooski, VT, USA) at 570 nm with blank corrections. The cell viability was calculated with respect to the control cells:

(1) $$Cell\;viability\;\left(\%\right)=\left[\left(Abs_{sample}-Abs_{DMSO}\right)/\left(Abs_{control}-Abs_{DMSO}\right)\right]\times 100$$

where $Abs_{sample}$ is the absorbance of the sample, $Abs_{DMSO}$ is the absorbance of the DMSO solvent and $Abs_{control}$ is the absorbance of the control.

### 2.9. Cellular Uptake Assay

Cells were seeded in 24-well plates at the concentration of 138,320 cells/well and then incubated for 24 h at 37 °C and 5% CO_2_, for cell adherence. The culture medium was then replaced with 500 µL of growth medium with pristine CNCs and CNCs/FA-CS-FITC nanosystems at 200 µg mL^−1^, and the cells were incubated with the previously described culture conditions. Acellular medium and control cells (no treatment) were also incubated. After the 24 h incubation period, the medium was collected and 400 µL aliquots were stored at −80 °C. Following washing with 250 µL of PBS, the cells were trypsinized and 250 µL of growth medium was added to neutralize trypsin. Two parameters, namely side-scattered light, and side-fluorescence light (excitation at 488 nm and measurement with a 530/30 band pass filter), were measured in an Attune^®^ Acoustic Focusing Cytometer (ThermoFisher Scientific, Waltham, MA, USA). At least 50,000 cells were examined for each test. The data were analyzed by FlowJo software (FlowJo LLC, Ashland, OR, USA).

### 2.10. Cellular Exometabolomics

For the NMR analysis, the medium samples collected during the cellular uptake assay (described in Section 2.9) were processed to remove interfering proteins. Briefly, 700 µL of cold methanol were added to 350 µL of medium, followed by 30 min resting at −20 °C, centrifugation (13,000× *g*, 20 min) and vacuum drying of the supernatant. Dried samples were then reconstituted in 600 µL of deuterated PBS (100 mM, pH 7.4) and transferred into 5 mm NMR tubes.

The NMR spectra were acquired on a Bruker Avance III HD 500 NMR spectrometer (Bruker Corporation, Billerica, MA, USA) operating at 500.13 MHz for ^1^H observation using a 5 mm TXI probe. Standard 1D ^1^H spectra with water pre-saturation (pulse program ‘noesypr1d’, Bruker library) were recorded with 32 k points, 7002.801 Hz spectral width, a 2 s relaxation delay and 512 scans. Spectral processing (TopSpin 4.0.3, Bruker BioSpin) comprised cosine multiplication (ssb 2), zero-filling to 64 k data points, manual phasing, baseline correction, and calibration to the TSP-*_d4_* signal (δ 0 ppm). Selected signals representative of the main metabolites detected were then integrated (Amix-Viewer 3.9.15, Bruker Biospin) and, for each metabolite, the fold change relative to the acellular medium was calculated for control and exposed groups to assess the magnitude of consumptions/secretions.

### 2.11. Statistical Analysis

Cellular viability, uptake and exometabolomics data were analyzed using the GraphPad Prism Software (GraphPad Software Inc., San Diego, CA, USA) with the data presented as the mean values ± standard error. Where differences existed, the source of the differences at the *p* < 0.05 significance level was identified by all pairwise multiple comparison procedures via the Tukey’s test.

## 3. Results and Discussion

**Figure 1:**
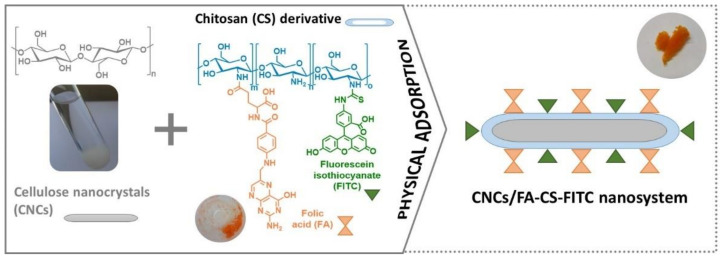
Scheme representing the preparation of the CNCs/FA-CS-FITC nanosystems.

Nanosystems based on cellulose nanocrystals (CNCs) and a multifunctional chitosan (CS) derivative were prepared via the simple (and eco-friendly) physical adsorption of the CS derivative, containing targeting (folic acid, FA) and imaging (fluorescein isothiocyanate, FITC) ligands (i.e., FA-CS-FITC), on the surface of the CNCs (Figure 1). Herein, the FA vitamin was carefully chosen as a targeting agent given the over-expression of folate receptors (FRs) in several tumor cells (and under-expression in non-tumor cells) [9,33], while the FITC fluorophore was picked for being a widely used imaging probe for the flow cytometry assays [33]. Furthermore, the CNCs were selected for their elongated nanostructure and anionic surface charge [29], whereas the CS derivative was chosen for its imaging and targeting moieties, as well as the cationic surface charge due to the presence of the characteristic protonated amine groups of CS at an acidic pH [34]. The assembly of the CNCs/FA-CS-FITC nanosystems via non-covalent interactions was preferred to covalent bonding due to the milder reaction conditions, when compared with previous studies where the functionalization of the CNC nanosystems was performed with fairly toxic reagents [20,22,23,25].

The opposite surface charge of these two polysaccharides enabled the facile physical adsorption [35] of the multifunctional CS derivative (i.e., FA-CS-FITC) on the surface of the elongated CNCs (Figure 1). The CNCs/FA-CS-FITC nanosystems were characterized in terms of structure, optical properties, fluorescence, stability in simulated physiological and non-physiological conditions, morphology, in vitro cytotoxicity, cellular internalization, and exometabolomics profile to evaluate their suitability for the targeting and imaging of breast cancer cells.

### 3.1. Preparation and Characterization of the CNCs/FA-CS-FITC Nanosystems

The synthetic pathway to obtain the CNCs nanosystems with targeting and imaging functions (Figure 1) comprised three main steps. First, the CNCs were extracted from cellulose cotton fibers via acid hydrolysis with phosphotungstic acid [29]. Then, the FA-CS-FITC derivative was synthesized via a two-step pathway, where the CS was initially functionalized with FA by a carbodiimide-mediated amidation reaction, followed by the reaction of this intermediate with the isothiocyanate groups (N=C=S) of the FITC to obtain the FA-CS-FITC derivative [30,31]. Lastly, the simple and eco-friendly physical adsorption of the FA-CS-FITC derivative (ζ-potential of +51.9 ± 3.6 mV at pH 3) on the surface of the CNCs (ζ-potential of −11.5 ± 0.7 mV at pH 7) generated the CNCs/FA-CS-FITC nanosystems (Figure 1). Herein, three CNCs nanosystems with different contents of the FA-CS-FITC derivative were prepared, namely CNCs/FA-CS-FITC_1 with a content of 13 μg of FA-CS-FITC per mg of CNCs, CNCs/FA-CS-FITC_2 with 29 μg mg^−1^ and CNCs/FA-CS-FITC_3 with 59 μg mg^−1^ (Table 1). The adsorption efficiencies of the FA-CS-FITC derivative on the surface of the CNCs (determined by UV-vis spectroscopy) were ca. 64% for CNCs/FA-CS-FITC_1, and around 72% for both CNCs/FA-CS-FITC_2 and CNCs/FA-CS-FITC_3 (Table 1).

**Figure 2:**
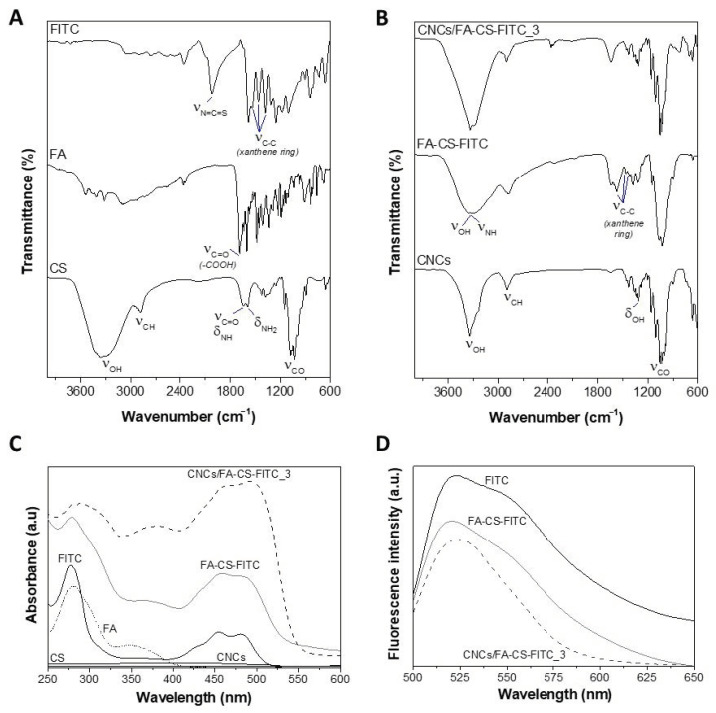
(**A**) FTIR-ATR spectra (vibrational modes: *ν* = stretching, *δ* = bending) of the pure CS, FA and FITC, (**B**) FTIR-ATR spectra of the pristine CNCs, FA-CS-FITC derivative and CNCs/FA-CS-FITC_3 nanosystem, (**C**) UV-vis spectra of CNCs, CS, FA, FITC, FA-CS-FITC derivative and CNCs/FA-CS-FITC_3 nanosystem, and (**D**) fluorescence emission spectra of the FITC, FA-CS-FITC derivative and CNCs/FA-CS-FITC_3 nanosystem.

The first indication of the successful assembly process between the CNCs and the CS derivative was given by the color change of the elongated CNCs from white to an orange color, as illustrated in Figure 1. This was further corroborated by FTIR-ATR, UV-vis, and fluorescence spectroscopy, as exemplified in Figure 2 for the nanosystem with the higher content of the FA-CS-FITC derivative, i.e., CNCs/FA-CS-FITC_3 (Table 1).

The FTIR-ATR spectrum of the CNCs/FA-CS-FITC_3 nanosystem (Figure 2B) presents predominantly the characteristic vibrations of the CNCs at 3342 cm^−1^ (O–H stretching), 2902 cm^−1^ (C–H stretching), 1316 cm^−1^ (O–H in-plane bending), and 1032 cm^−1^ (C–O stretching) (Figure 2B) [36], but also those of the CS derivative, namely from: (i) chitosan at 3342 cm^−1^ (O–H and N–H stretching), 1636 cm^−1^ (C=O stretching and N–H bending), 1592 cm^−1^ (–NH_2_ bending), 1380 cm^−1^ (–CH_2_ bending), 1078 and 1032 cm^−1^ (C–O stretching) [37]; (ii) folic acid at 1688 cm^−1^ (C=O stretching), 1602 and 1481 cm^−1^ (C=C aromatic) cm^−1^ (phenyl ring) [38], and (iii) FITC at 1545, 1458 and 1378 cm^−1^ (xanthene ring skeletal C–C stretching) [39,40,41], as depicted in Figure 2A. However, the majority of these absorption bands overlap because of the common functional groups of the individual components (i.e., CNCs, CS, FA and FITC). Worth noting here is the absence of the absorption bands at 1686 cm^−1^ (carboxyl moiety of FA [38]) and 2018 cm^−1^ (isothiocyanate moiety (N=C=S) of the FITC [39]), together with the appearance of the absorption band (ca. 1650 cm^−1^) assigned to the amide thioamide bonds [42], that confirm the covalent link between CS and FA [43], and CS and FITC [44], respectively, in agreement with data reported elsewhere [45].

The UV-vis spectrum of the CNCs/FA-CS-FITC_3 nanosystem (Figure 2C) exhibits only the characteristic bands of the CS derivative, given that the CNCs do not present any absorption in the UV and visible regions, as registered elsewhere [20,46]. On the other hand, the bands of the FA-CS-FITC derivative (Figure 2C) are typical of the FA (at ca. 285 and 356 nm ascribed to the π–π* and n–π* transitions, respectively [38]) and FITC (at about 289, 462 and 490 nm [39,40]) ligands since the pure CS polysaccharide does not display any absorption in the UV and visible regions [47]. Predictably, some of the absorption bands were red-shifted compared to those reported for the pure FA and FITC, which is an indication of the covalent bond between CS, FA and FITC in the FA-CS-FITC derivative, as described for other systems such as FA-polyaniline [38] and FITC-organoclay [39].

The fluorescence emission spectrum of the CNCs/FA-CS-FITC_3 nanosystem (Figure 2D) shows an absorption maximum at ca. 524 nm, which mimics the emission fluorescence peak of the FA-CS-FITC derivative. This peak is characteristic of the FITC fluorophore that, as reported by Ghosh et al. [40], upon excitation at a wavelength of 490 nm presents a strong emission peaking at around 520 nm. As anticipated, the pristine CNCs showed no emission in the wavelength range of the visible region. These results indicate that the CNCs nanosystems have fluorescent properties since the FITC fluorophore maintained its fluorescence. This behavior was expected given that the FITC is widely used to attach a fluorescent label mostly to polysaccharides (e.g., FITC-labelled CS [33,48], FITC-labelled CNCs [20,49], FITC-labelled dextran [40]) and proteins (e.g., FITC-labelled albumin [50]).

The success of the assembly process was additionally confirmed by testing the stability of the CNCs/FA-CS-FITC_3 nanosystem under simulated non-physiological and physiological pH conditions, namely pH 2.1 for acidic medium [32] and pH 7.2 for human blood plasma [51], respectively. Since these CNC nanosystems rely on the physical adsorption between the negatively charged CNCs and the cationic CS derivative, it is important to assess the potential release of FA-CS-FITC from the nanosystems to avoid ambiguous interpretations of the results or even unnecessary cytotoxic effects, which might hinder their applicability [52]. According to the obtained data, the amount of the CS derivative released from the CNCs/FA-CS-FITC_3 nanosystem is low at a non-physiologic pH with values of 6.6 ± 0.8% and 8.4 ± 0.5% for 24 h and 48 h, respectively. At the physiological pH 7.2, the release was slightly higher, with values of 11.0 ± 0.1% and 14.5 ± 0.1% for 24 h and 48 h, respectively. Overall, the CNCs/FA-CS-FITC nanosystems present good stability and dispersibility in both pH conditions, which can be credited to the effective electrostatic interactions established between the anionic CNCs and the cationic FA-CS-FITC derivative.

**Figure 3:**
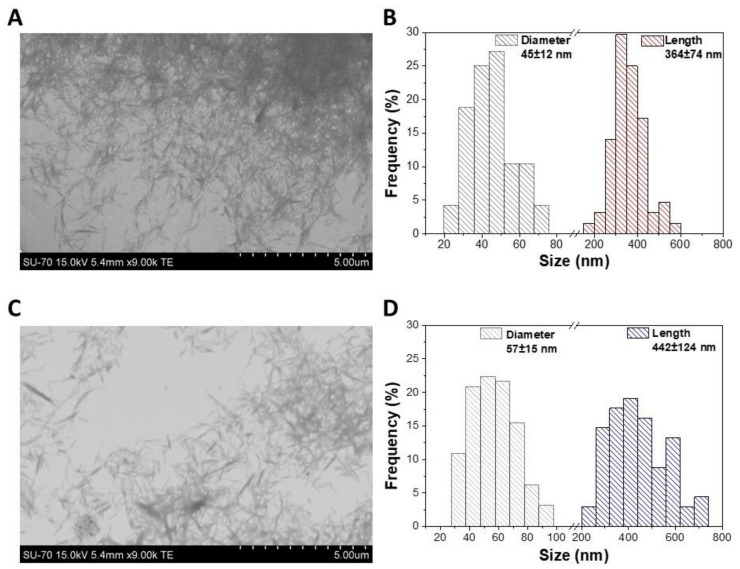
STEM micrographs (**A**,**C**) with the corresponding size histograms (diameter and length, (**B**,**D**)) of the (**A**,**B**) pristine CNCs and (**C**,**D**) CNCs/FA-CS-FITC_3 nanosystem.

The morphology and size of the CNCs nanosystems functionalized with the FA-CS-FITC derivative were assessed by STEM (Figure 3). As anticipated, the pristine CNCs showed the typical rod-like morphology with dimensions of 45 ± 12 nm (diameter) and 364 ± 74 nm (length), analogous to the data described in the literature [29]. After the assembly process, the CNCs maintained the rod-like morphology, but their dimensions slightly increased to 57 ± 15 nm (diameter) and 442 ± 124 nm (length). This minor increase in the size range is clearly a direct result of the physical adsorption of the CS derivative on the surface of the CNCs, and it was also observed for other CNCs-based nanosystems [25,53].

Both the shape and size of the CNCs/FA-CS-FITC nanosystems are parameters of utmost importance due to the cell-size-dependent uptake of nanoparticles by the cells [14,17]. Thus, one can speculate that the elongated morphology of the CNCs/FA-CS-FITC nanosystems, together with their nanometric size, will not hinder the cellular internalization by the FR-positive breast cancer cells (i.e., MDA-MB-231 cell line), as will be discussed in the following paragraphs.

### 3.2. Cellular Viability

The in vitro cytotoxicity of the pristine CNCs and the CNCs/FA-CS-FITC nanosystems was evaluated in human breast adenocarcinoma cells (i.e., MDA-MB-231 cells) for 24 h at concentrations ranging from 12.5 to 200 µg mL^−1^ using the indirect MTT assay. The MDA-MB-231 cell line was selected for being often used as a model of triple-negative breast cancer cells [54] with over-expression of folate receptors [55], and for which the available therapeutic options are more scarce than other breast cancer subtypes [56].

**Figure 4:**
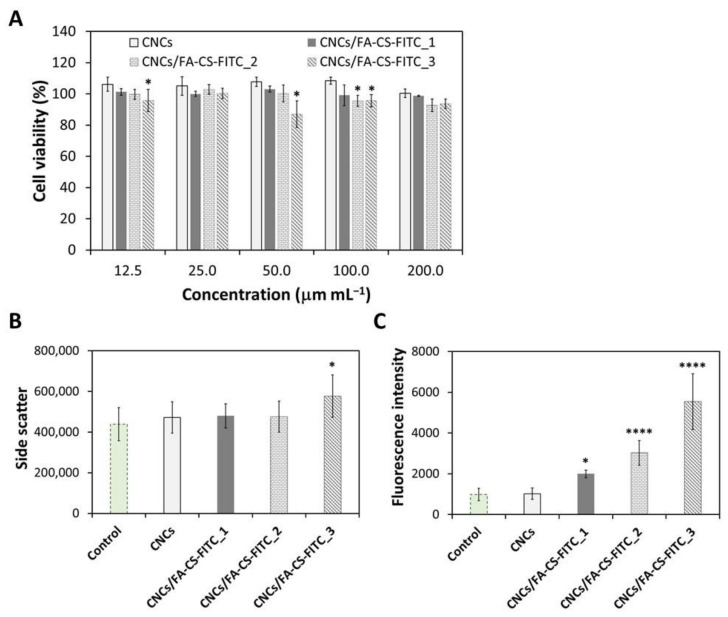
(**A**) Cell viability of the MDA-MB-231 cells after 24 h of exposure to the pristine CNCs and CNCs/FA-CS-FITC nanosystems, and flow cytometry data: (**B**) side-scattered light and (**C**) side-fluorescence light of the pristine CNCs and CNCs/FA-CS-FITC nanosystems for 24 h (the symbols * and **** epitomize the means with a significant difference from the control at *p* < 0.05 and *p* < 0.0001 levels, respectively).

According to the data presented in Figure 4A, the cell viability of the MDA-MB-231 cells after 24 h of exposure to the pristine CNCs at five different concentrations (12.5, 25, 50, 100 and 200 µg mL^−1^) remained at the level of 100%, meaning that the cell viability is not dose-dependent in the tested concentration range. This outcome for the pristine CNCs is consistent with the results found in the literature for this cell line [22], but also for other FR-positive human cancer cell lines, e.g., MDA-MB-468, KB, and PC-3 cells [22].

Regarding the CNCs/FA-CS-FITC nanosystems, the profile is the same and the cell viability is higher than 90% for all the tested concentrations. This confirms without a doubt that the three CNCs/FA-CS-FITC nanosystems are non-cytotoxic to the MDA-MB-231 cells at concentrations below 200 µg mL^−1^, with an in vitro cell viability quite above the 70% threshold [57]. These results are similar to those reported for other CNCs-based nanosystems with other tumor cell lines, such as the human breast adenocarcinoma MCF-7 cell line [58], and the A375 and M14 cells (malignant melanoma cell lines) [59].

The non-cytotoxicity of the three CNCs/FA-CS-FITC nanosystems for the tested concentration range evinces their potential to act as nanocarriers of imaging and therapeutic agents for targeted therapy, without inhibiting the viability of the FR-positive breast cancer cells. In this sense, the cellular internalization assays will be performed with the highest tested concentration, namely at 200 µg mL^−1^ of each of the CNCs/FA-CS-FITC nanosystems, as discussed in the following paragraphs.

### 3.3. Cellular Internalization

The in vitro cellular internalization of the CNCs/FA-CS-FITC nanosystems by the MDA-MB-231 cells was studied by flow cytometry (side-scattered light (Figure 4B) and side-fluorescence light (Figure 4C)) for an exposure time of 24 h. The data compiled in Figure 4B shows that the incubation of the MDA-MB-231 cells in the presence of the pristine CNCs did not result in cell internalization, which is in line with the findings reported for this cell line [22], but also for other FR-positive human cancer cell lines, namely KB and MDA-MB-468 cells [22,24,25], and PC-3 cells [22].

In the case of the CNCs/FA-CS-FITC nanosystems, the ones with the lower content of the CS derivative, i.e., CNCs/FA-CS-FITC_1 (13 μg of FA-CS-FITC per mg of CNCs) and CNCs/FA-CS-FITC_2 (29 μg of FA-CS-FITC per mg of CNCs), showed a minor enhancement when compared to the control, and thus the cell internalization was minimal. In fact, only the CNCs/FA-CS-FITC_3 nanosystem (59 μg of FA-CS-FITC per mg of CNCs) was significantly internalized by the FR-positive breast cancer cell line (*p* < 0.05).

Since these measurements are proportional to cell granularity or cell complexity and thus can be sometimes less sensitive [60], the fluorescence intensity of the cell population was also evaluated (Figure 4C) by taking advantage of the existent FITC fluorophore probe in the CNCs/FA-CS-FITC nanosystems. Once again, the cells incubated in the presence of the pristine CNCs exhibited a fluorescence intensity matching the control, which agrees with the results shown in Figure 4B. On the contrary, the CNCs/FA-CS-FITC nanosystems displayed a significant increase in the fluorescence intensity (Figure 4C). Basically, it is possible to observe the augment of the fluorescence intensity with the increasing content of the CS derivative (i.e., FA-CS-FITC, Table 1), reaching a maximum 5-fold increase compared to the control. The growing cell internalization by the FR-positive breast cancer cells is credited to the increasing content of FA.

Notably, this outcome also validates that the size of the CNCs/FA-CS-FITC nanosystems (Figure 3) did not hamper in any way the effective internalization of these FA-target nanosystems by the FR-positive breast cancer cells, as is in fact shown with other CNCs-based nanosystems with FA ligands [23,25,26].

### 3.4. Cellular Exometabolomics

**Figure 5:**
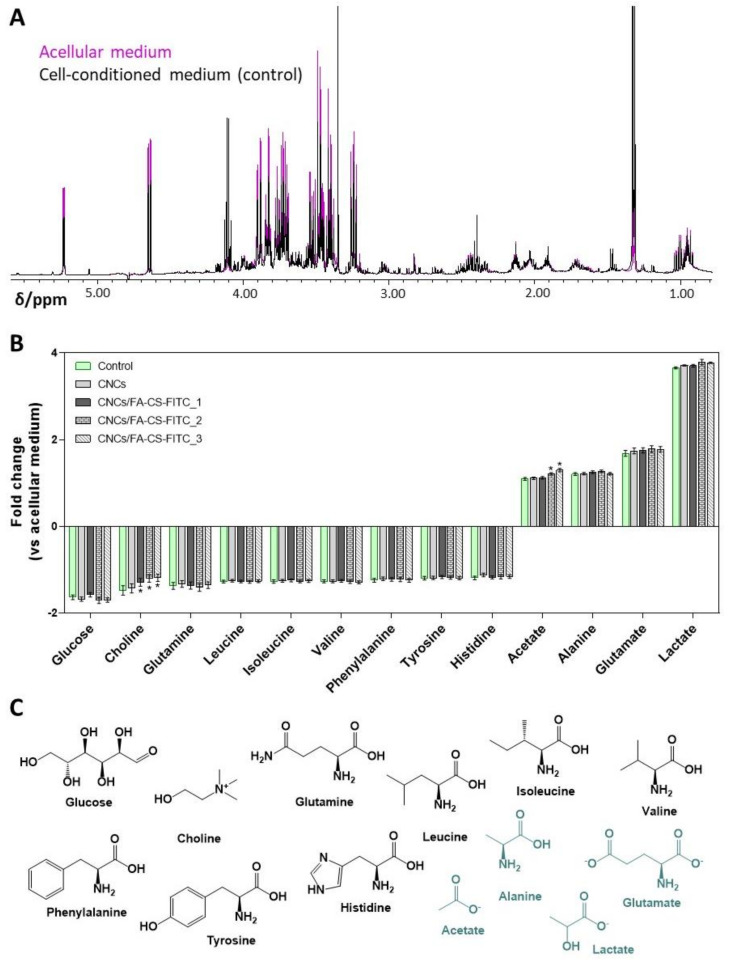
(**A**) ^1^H NMR spectra of the acellular medium (pink) and supernatant from MDA-MB-231 breast cancer cells grown for 24 h (black), (**B**) variations in metabolites consumed (negative bars) and excreted (positive bars) by the MDA-MB-231 cells, under control conditions and upon treatment with 200 μg mL^−1^ of CNCs, CNCs/FA-CS-FITC_1, CNCs/FA-CS-FITC_2 and CNCs/FA-CS-FITC_3 for 24 h (the symbol * represents the means with a significant difference from the control at *p* < 0.05), and (**C**) chemical structures of the consumed (black) and excreted (green) metabolites.

The extracellular metabolomic profile of the MDA-MB-231 cells was studied by ^1^H NMR spectroscopy to assess possible changes in the cells metabolic activity induced by the 24 h exposure to the pristine CNCs and the CNCs/FA-CS-FITC nanosystems (200 µg mL^−1^). Figure 5A shows the characteristic ^1^H NMR spectra of the MDA-MB-231 cell-conditioned medium and of the acellular culture medium incubated under identical conditions. Integration of the NMR signals representative of the main metabolites allowed the determination of the metabolite consumption and secretion by control and exposed cells, as summarized in Figure 5B.

The MDA-MB-231 control cells mainly consumed glucose and choline, along with some amino acids (glutamine, branched-chain amino acids, aromatic amino acids, and histidine), while excreting lactate, glutamate, alanine, and acetate (Figure 5B,C). These findings are similar to those reported by Guerra et al. [61] and highlight the fact that the MDA-MB-231 cell line, like most cancer cells, exhibits the classical Warburg effect with a high glucose uptake and lactate production, together with high glutaminolytic activity [62].

Upon exposure of the MDA-MB-231 cells to the CNCs, there were no changes in their metabolic activity, which is not unexpected, given that the pristine CNCs were poorly internalized (Figure 4B,C). However, when cells were incubated with the CNCs/FA-CS-FITC nanosystems, slight but significant dose-dependent differences were found in choline uptake and acetate secretion (Figure 5B). The CNCs functionalized with intermediate and high FA concentrations (i.e., CNCs/FA-CS-FITC_2 and CNCs/FA-CS-FITC_3, respectively) caused a decrease in choline consumption and an increase in acetate release to the extracellular medium. Choline is an essential vitamin-like nutrient required for the de novo synthesis of membrane phospholipids, like phosphatidylcholine and sphingomyelin [63]. Various types of cancer cells, including breast cancer, display enhanced choline uptake and altered metabolism, to support fast proliferation and migratory capacity [64]. Hence, it is possible that the herein observed decrease in choline consumption by the cells incubated with the CNCs/FA-CS-FITC nanosystems reflects a lower synthesis of membrane lipids and a slower proliferation. This is consistent with the observed increase in the amount of acetate released by cells upon incubation with the nanosystems containing the highest concentrations of the FA-CS-FITC derivative (i.e., CNCs/FA-CS-FITC_2 and CNCs/FA-CS-FITC_3, Table 1). Indeed, besides glucose and glutamine, acetate provided as an extracellular nutrient or produced endogenously from pyruvate [65], may chiefly contribute to de novo lipid synthesis via conversion into acetyl-CoA by cytosolic and/or mitochondrial acetyl-CoA synthetases [66]. Hence, its release into the culture medium could reflect its lower intracellular utilization, possibly in relation to the downregulation of lipogenesis.

To summarize, the assembly of the CNCs/FA-CS-FITC nanosystems via physical adsorption was inspired by the possibility of using the opposite surface charge of the CNCs and the FA-CS-FITC derivative and originated non-cytotoxic nanosystems up to concentrations of 200 µg mL^−1^. Furthermore, and since the cellular uptake is a limiting factor for the efficacy of countless anticancer drugs, the presence of FA in the CNCs/FA-CS-FITC nanosystems promoted a higher cellular internalization towards FR-positive MDA-MB-231 breast cancer cells. Besides, mild alterations in the cells’ exometabolome upon 24 h exposure to the CNCs/FA-CS-FITC nanosystems suggest an anti-proliferative effect, which may be beneficial in the context of cancer treatment.

Henceforth, the CNCs/FA-CS- FITC nanosystems can be exploited as nanocarriers of imaging and chemotherapeutics agents for active targeted therapy. In fact, the incorporation of, for instance, gold nanoparticles (with potential for photothermal cancer therapy [67]) into the CNCs/FA-CS-FITC nanosystems could be an option to engineer a nanocarrier with combined diagnostic and therapeutic capabilities, viz. a theranostic nanosystem [68].

## 4. Conclusions

In the present work, nanosystems composed of CNCs and a CS derivative were successfully developed and characterized. The physical adsorption of the CS derivative, containing a targeting ligand (FA) and an imaging agent (FITC), on the surface of the CNCs was an eco-friendly methodology to obtain CNCs-based nanosystems. The ensuing nanosystems displayed good stability in two distinct simulated non-physiological and physiological conditions (pH 2.1 and 7.2, respectively), as well as non-cytotoxicity towards MDA-MB-231 cells up to a concentration of 200 µg mL^−1^. The CNCs nanosystems showed a superior cellular internalization with a 5-fold increase in the fluorescence intensity for the nanosystem with the greater content of FA, viz. concentration-dependent internalization by the FR-positive breast cancer cells. Additionally, the exometabolomics of MDA-MB-231 cells exposed to the CNCs/FA-CS-FITC nanosystems revealed a mild impact on the metabolic activity of the cells, namely a decreased choline uptake and increased acetate release, which suggests an anti-proliferative effect. The overall data evidenced that the elongated CNCs/FA-CS-FITC nanosystems produced by an eco-friendly methodology have high affinity towards folate receptor-positive cancer cells with enhanced cellular internalization, and hence might be employed as nanocarriers with imaging properties for active targeted therapy.

## Figures and Tables

**Table 1:** Composition of the prepared CNCs/FA-CS-FITC nanosystems.

Nanosystems	Nominal Composition ^a^		Measured Composition ^b^	
	W_CNCs_ (mg)	W_FA-CS-FITC_ (mg)	W_FA-CS-FITC_ (mg)	W_FA-CS-FITC_/W_CNCs_
CNCs/FA-CS-FITC_1	50.0	1.01	0.65	0.013
CNCs/FA-CS-FITC_2	50.0	1.99	1.43	0.029
CNCs/FA-CS-FITC_3	50.0	4.09	2.97	0.059

^a^ The nominal composition is the initial mass of CNCs (W_CNCs_) and CS derivative (W_FA-CS-FITC_). ^b^ The measured composition was indirectly determined by measuring the absorbance of the washing solutions of each nanosystem at 441 nm (calibration curve: $y=2.2591x-0.0141,\;R^{2}=0.9995$), concentration range of the FA-CS-FITC derivative: 0.005–0.5 mg mL^−1^).
